# Feasibility of an Unguided, Parent-Focused, Online Program for Sleep Problems in Young Children: The Lights Out Online Program

**DOI:** 10.1007/s10578-025-01837-z

**Published:** 2025-04-23

**Authors:** Caroline L. Donovan, Evren Etel, Laura Uhlmann, Amy Shiels, Tamsin Joynt, Sonja March, Lisa J. Meltzer, Laurie McLay, Lara J. Farrell, Allison M. Waters, Robert S. Ware, Annie Storey

**Affiliations:** 1 School of Applied Psychology, Griffith University, Brisbane, Gold Coast, QLD Australia; 2 Griffith Centre for Mental Health, Griffith University, Gold Coast, Brisbane, Australia; 3 Centre for Health Research, School of Psychology and Wellbeing, University of Southern Queensland, Queensland, Australia; 4 Department of Pediatrics, National Jewish Health, Denver, CO USA; 5 Child Well-Being Research Institute, Faculty of Health, University of Canterbury, Christchurch, New Zealand

**Keywords:** eHealth, Pediatric insomnia, Behavioural intervention, Child, Parent

## Abstract

Sleep problems in young children are highly prevalent and place children at risk for numerous detrimental child and family outcomes. This pilot study aimed to assess the feasibility of an unguided, parent-focused, online sleep intervention, the Lights Out Online program, in terms of adherence rates, acceptability to parents, and effects on (a) child sleep, anxiety, and behaviour problems, and (b) parental self-efficacy, and parent sleep, depression, anxiety. The study was a pilot, open (uncontrolled) trial with a within-group repeated measures (baseline (T1), 12-weeks post-baseline (T2)) design. Participants were 24 parents *(M*_*age*_ = 36.2, *SD* = 4.1) of children aged 3–6 years (*M*_*age*_ = 4.1 years, *SD* = 1.1) with sleep problems. Participants completed an average of 3.58 out of 4 sessions by T2, and parents reported satisfaction with program content and presentation. A series of mixed-effects linear regression models demonstrated significant improvements in child sleep, child anxiety, child behaviour problems, parent sleep, parent anxiety and parental self-efficacy, from T1 to T2. However, significant improvements were not demonstrated for parent depression and parent stress. The results of this study should be taken with caution given the small sample size and lack of control group. However, the study provides preliminary support for the feasibility of Lights Out Online.

## Introduction

Sleep problems amongst young children aged 3–6 years are extremely common, with 38% of youngsters experiencing some kind of sleep difficulty (Quach et al. [60]), and 14% experiencing moderate to severe sleep dysfunction (Hiscock et al. [35]). Although some young children experience biologically-based sleep problems such as breathing problems (e.g., obstructive sleep apnoea) and parasomnias (e.g., sleep walking and night terrors, and sleep paralysis), behavioural sleep problems are by far the most common, afflicting 30% of youngsters (Meltzer et al. [47]). Defined as sleep difficulties that cannot be causally attributed to internal physical processes, behavioural sleep problems include difficulties initiating and maintaining sleep, bedtime resistance, and difficulties sleeping without parental presence or assistance (Hannan and Hiscock [30]).

The high prevalence of sleep problems in young children is concerning given the rapid brain maturation that occurs during this time, and the essential role that sleep plays in restorative processes, consolidation of learning and memory, and facilitation of somatic and neurological growth (Gómez and Edgin [24]); Reynaud et al. [62]. Indeed, sleep problems in young children have been shown to be associated with a vast array of detrimental sequela including poorer academic performance and competence (Hoyniak et al. [38]); Paavonen et al. [58], greater working memory deficits (Nelson et al. [55]), poorer language, mental ability, and fine motor skills (Yorbik et al. [85]), and poorer executive function and attention regulation (Bernier et al. [5]); Reynaud et al. [62]; Williams et al. [84]. Sleep problems have also been found to affect young children socially, being associated with poorer social skills (Yorbik et al. [85]), less mature empathy styles (Rong et al. [65]), less peer acceptance, poorer social competence and less social engagement (Vaughn et al. [80]). Of all the consequences with which sleep problems have been associated with in young children however, their relationship with anxiety and behaviour problems are among the strongest.

With respect to anxiety, preschool sleep problems are not only concurrently associated (Ding et al. [15]), but have also been shown to predate anxiety problems. Indeed, preschool sleep problems have been shown to predict anxiety at 7 years of age (Gregory et al. [26]), during primary school (Whalen et al. [83]), throughout adolescence (Gregory and O’Connor [25]), and into adulthood (Gregory et al. [25]). Similarly, preschool sleep problems are not only cross-sectionally related to fewer positive behaviours, and more behavioural, externalising and aggression difficulties (Bates et al. [4]); Hiscock et al. [35]; Quach et al. [61], but have also been shown to predict fewer prosocial behaviours one year later (Hatzinger et al. [32]), as well as more conduct problems and hyperactivity at 7 years of age (Gregory et al. [26]), and greater aggression during adolescence (Wang et al. [81]).

In addition to the myriad child-related problems associated with sleep difficulties in young children, parents are also affected. Indeed, parents of young children with sleep problems report greater variability in their own bed time (Varma et al. [79]), higher pre-sleep arousal (Varma et al. [78]), lower sleep quality (Chehri et al. [12]); Roberts et al. [63], shorter sleep duration (Carroll et al. [10]), and greater fatigue and daytime sleepiness compared to parents of children without sleep problems (Meltzer and Mindell [47]); Roberts et al. [63].

In addition to parent sleep problems, child sleep problems have also been associated with poorer parental mental health, especially for mothers, across depression, anxiety and stress (Amaerjiang et al. [2]); Schultz et al. [68]; Tyler et al. [76]; Ystrom et al. [86]). A cross-sectional survey of Australian children in their first year of school (*N* = 1512, *M*_*age*_ = 5.7 years, *SD* = 0.4) found a strong association between parent reported child sleep problems and parental mental health, with even mild child sleep problems being associated with higher parental anxiety, depression and stress (Quach et al. [60]).

Finally, general parental self-efficacy is also affected by child sleep problems. Defined as a parent’s belief in their own ability to effectively manage parental tasks, parental self-efficacy taps into parental self-esteem, parenting confidence, and subjective competence (Vance and Brandon [77]). It has been found that fewer child sleep problems (Fang et al. [21]); Werner et al. [82], longer child sleep duration (Heerman et al. [33]) and fewer child sleep disturbances are observed when parents exhibit higher general parenting self-efficacy (Kim et al. [39]). It has been suggested that parents who have difficulty effectively managing their young child’s sleep problems lose confidence in their parenting abilities, leading to negative self-appraisal and lower self-esteem (Werner et al. [82]). Indeed, helping parents to effectively manage their child’s sleep problems has been proposed as a means to improve parental self-efficacy (Carroll et al. [10]).

Although for some young children sleep problems are transient and remit naturally over time, for others sleep problems persist into older childhood and adolescence if left untreated (Falch-Madsen et al. [20]); Simola et al. [69]; Wang et al. [81]. Given the high prevalence rates and problematic consequences associated with preschool sleep problems, it is of some reassurance that we can treat them well. Several meta-analyses and reviews have concluded that behavioural interventions for behavioural sleep problems in young children are efficacious (Fangupo et al. [22]); Meltzer and Mindell [47]; Meltzer et al. [50]; Mindell [51]. Interestingly however, the majority of studies included within these reviews have not included preschool age children within their samples, and have instead focused on infants and toddlers. Furthermore, despite the demonstrated deleterious impact of sleep problems in young children across a wide range of emotional, social and cognitive outcomes, the majority of treatment studies that have targeted young children have not measured outcomes other than sleep (Meltzer et al. [50]), and when they have, the results have been inconsistent and/or mixed (e.g., Hiscock et al. [36]); Quach et al. [59].

Recently, Donovan et al. [17] conducted a randomised controlled trial (RCT) of the Lights Out program, a parent-focused, face-to-face, group-based behavioural sleep program for preschool-aged children. It was found that the program was not only successful in reducing sleep problems in young children relative to a care as usual group, but that it also led to significant improvements in child anxiety, behaviour problems, and internalising and externalising behaviours. Furthermore, the authors were able to demonstrate that it was the improvement in child sleep problems that led to the improvements in child anxiety, internalising and externalising behaviours. Donovan et al. [17] attribute the broader emotional and behavioural effects of *Lights Out* to the inclusion of gold standard treatments for two of the most common behavioural sleep problems among preschool children, namely anxiety at night-time and behavioural problems at bedtime. Although standard sleep programs include treatment strategies such as psychoeducation, sleep hygiene, bedtime routines, and relaxation (Meltzer et al. [50]), they typically do not include exposure therapy for night-time fears, behaviour management for misbehaviour at bedtime, or parent training in how to positively reinforce children for good and/or brave behaviour. The Lights Out program includes these additional program components, equipping parents to better manage child anxiety and/or behaviour problems at bedtime and in contexts other than bedtime.

Despite its efficacy and broad impact on outcomes other than sleep, the Lights Out program is unlikely to be taken up in its current face-to-face, group-based delivery format. Only 11.1% of children with sleep problems seek treatment (Newton et al. [56]) due to numerous barriers to psychological help-seeking (Tapp et al. [75]). One way to circumvent these barriers is to employ an online delivery format, so that programs are accessible to all families with a computer and internet access. In Australia, where the Lights Out program has been developed, 97% of all households with a child under 15 years of age have access to the internet despite vast rural and remote areas. An online version of the program would therefore be a highly accessible treatment format for Australian families (Australian Bureau of Statistics [3]).

Internet-based programs have demonstrated efficacy across a range of childhood difficulties including anxiety (Donovan and March [16]); March et al. [45]; Spence et al. [73] and behavioural problems (Florean et al. [23]), and have been shown to be both acceptable and appealing to parents with the caveat that they are developed and delivered by reputable providers (Hansen et al. [31]); MacKinnon et al. [44]. With respect to child sleep, there is evidence to suggest that online programs targeting infants and toddlers (Leichman et al. [41]); Mindell et al. [52], older children aged 4–12 years with Autism Spectrum Disorder (Roberts et al. [63]), and samples that have included (but were not limited to) preschool age children (Brandhorst et al. [8]); Corkum et al. [13]; Hiscock et al. [36]; Schlarb and Brandhorst [67] are effective in reducing sleep problems. However, given the unique developmental characteristics and sleep challenges experienced by young children, it is surprising to find that researchers have not yet investigated an online program for sleep problems targeting neurotypical preschool children specifically.

### The Current Study

This study examines the feasibility of an unguided, online version of the Lights Out program (“Lights Out Online”), assessing program adherence (number of sessions and time taken to complete the program), acceptability (parental satisfaction), and the program’s effects on child (sleep, anxiety, and behaviour problems) and parent (parental self-efficacy, anxiety, depression, stress and sleep) outcomes. For this small, open, feasibility pilot trial, it was hypothesised that: (1) adherence would be high; (2) the program would be highly acceptable to parents; (3) child sleep, anxiety and behaviour problems would significantly improve from baseline (T1) to 12-weeks post-baseline (T2) and; (4) parental self-efficacy, sleep, depression, anxiety and stress would significantly improve from T1 to T2.

## Method

### Design

This study was an open (uncontrolled) trial with assessments taken at baseline (T1) and 12-weeks post-baseline (T2). The study was conducted ahead of a larger, RCT that is currently being undertaken, and was designed to assess program feasibility, iron out program bugs, and garner feedback from parents to inform program modification and refinement. Given the aims of the present study, it was considered appropriate and prudent to employ an open trial with a small sample size and pre-post design. The 12-week post-assessment timepoint was chosen to (a) align with the primary endpoint in our previous *Lights Out* studies, and (b) to allow sufficient time for parents to complete the program given that previous trials of self-directed online programs targeting children have found participants take longer to complete sessions compared to face-to-face therapy (Spence et al. [72]).

**Table 1:** Demographic Characteristics of Participants (*N =* 24)

Variables	Range	*M (SD)*
Parent age (years)	28–46	36.2 (4.1)
Child age (years)	3–6	4.1 (1.1)
Number of children in household	1–4	2.0 (0.7)
*Parent*	*n*	%
Gender		
Female	23	95.8
Male	1	4.2
Relation to the child		
Mother	23	95.8
Father	1	4.2
Country of birth		
Australia	22	91.7
Europe	1	4.2
Asia	1	4.2
Relationship status		
Defacto	5	20.8
Married	19	79.2
Education level		
Grade 12 or below	2	8.3
TAFE/Trade Certificate	2	8.3
Diploma	2	8.3
Bachelor degree	11	45.8
Postgraduate degree	7	29.2
Employment		
Student	1	4.2
Full-time	11	45.8
Part-time	10	41.7
Not working	2	8.3
Household income		
$75,001 - $100,000	4	16.7
$100,001 - $125,000	3	12.5
$125,001 - $150,000	2	8.3
$150,001 - $175,000	5	20.8
More than $200,000	7	29.2
Prefer not to say	3	12.5
*Child*		
Gender		
Female	19	79.2
Male	5	20.8
Country of birth		
Australia	23	95.8
North America	1	4.2
Living arrangements		
Both biological parents	22	91.7
Biological mother only	1	4.2
Biological mother and another	1	4.2

### Participants

**Fig. 1:**
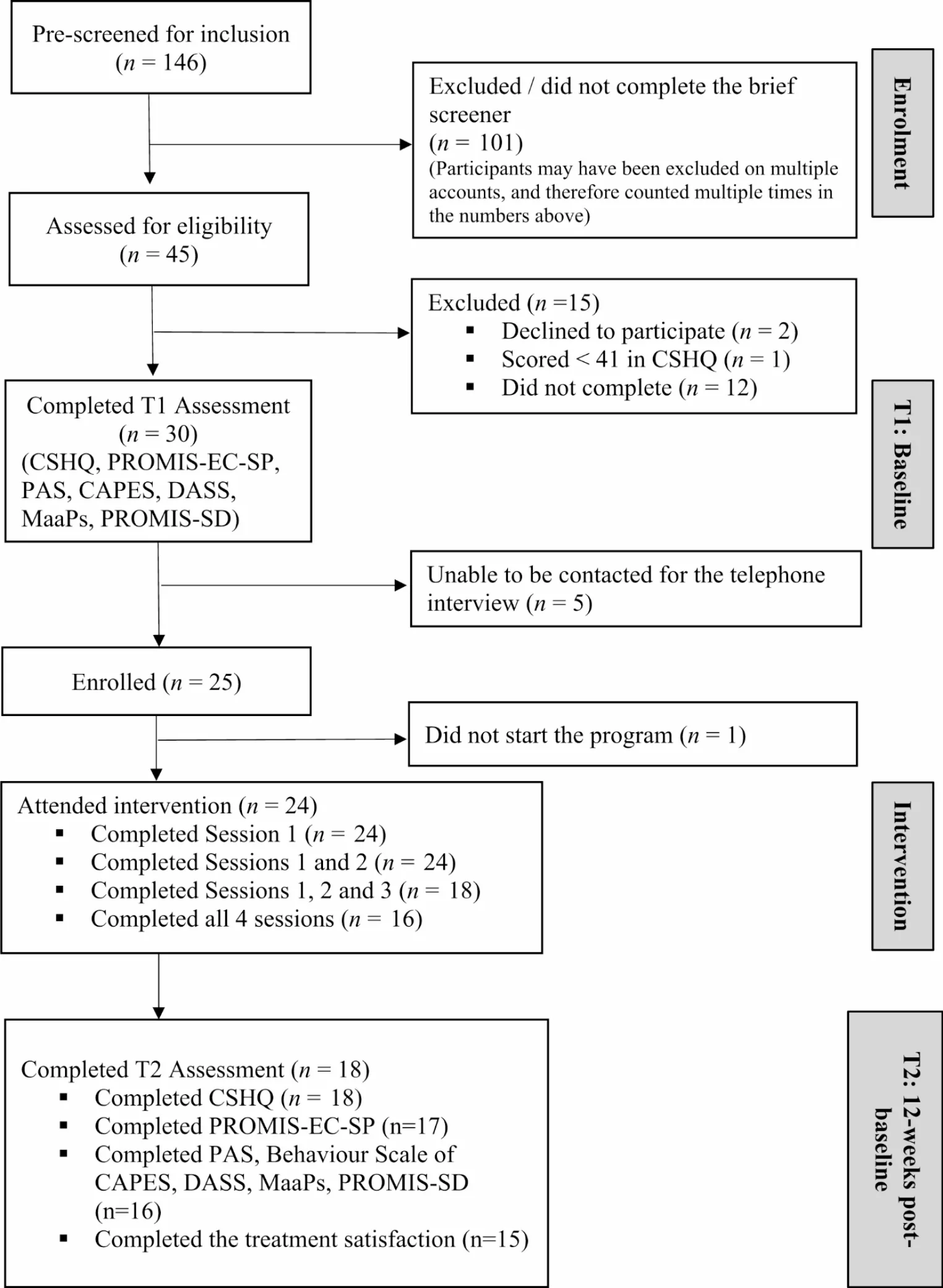
Flow of Participants Through the Study

Table 1 outlines the demographic characteristics of the sample and Fig. 1 outlines the flow of participants through the study. Participants were 24 parents *(M*_*age*_ = 36.2, *SD* = 4.1) of children aged 3–6 years (*M*_*age*_ = 4.1 years, *SD* = 1.1) who scored ≥ 41 on the *Child Sleep Habits Questionnaire* (CSHQ; Owens et al. [57]). Families were not eligible to participate if (a) the child had an intellectual or developmental disorder, (b) the parent was under 18 years of age, (c) the child had started/modified medication affecting/treating their sleep, (d) were currently receiving psychological assistance for the child’s sleep problem, and/or (e) the family resided outside of Australia.

### Measures

All measures were administered at T1 and T2, with the exception of the demographics questionnaire that was administered only at T1, and the satisfaction questionnaire that was administered only at T2.

#### Demographics

Parents were asked to report their own age, gender identity, relationship to child, country of birth, relationship status, education level, employment status, and household income. They were also asked to report their child’s age, gender, country of birth, living arrangements, and number of children in the household.

#### Program Adherence

Program usage data was tracked to provide data on the number of sessions parents completed and the number of weeks taken by parents to complete sessions. The average time to complete each session was calculated from when the session first became available to when the session was completed. Similarly, the average time to complete the program was calculated from the availability of Session 1 to the completion of Session 4 (or the final session completed).

#### Program Acceptability

Four items, previously used in open access, unguided, online, child anxiety programs (March et al. [45]); Spence et al. [71] were used to measure program satisfaction and acceptability at T2. Item 1, “*Would you tell a friend about this program if their child had sleep problems?”*, was rated on a scale from 1 (definitely would not) to 5 (definitely would). Item 2, “*How helpful was this program in assisting you to manage your child’s sleep problem?”* was rated on a scale from 1 (extremely unhelpful) to 5 (extremely helpful). Item 3, “*How happy were you with this program?”*, was rated on a scale from 1 (extremely unhappy) to 5 (extremely happy). Item 4, “Overall, I think this program was…”, was rated on a scale from 1 (extremely bad) to 5 (extremely good). Items were averaged to provide a mean total satisfaction and acceptability score that could range from 1 to 5, with higher scores reflecting greater satisfaction with, and acceptability of, the program.

#### Primary Outcome: Child Sleep

Child sleep was measured through both the Child Sleep Habits Questionnaire (CSHQ; Owens et al. [57]) and the PROMIS^®^ Early Childhood Parent Report Bank v1.0 0 Sleep Problems (PROMIS-EC-SP; Lai et al. [40]. The CSHQ is a 33-item parent-report measure assessing a range of child sleep problems. For 31 of the items, parents are required to rate on a 3-point scale from 1 (rarely; 0-1 times) through 2 (sometimes; 2–4 times) to 3 (usually; 5–7 times) the frequency with which each item applies to their child. For 2 of the items, parents are required to rate on a 3-point scale from 0 (not sleepy) through 1 (very sleepy) to 2 (falls asleep), how their child appears while (a) watching tv and (b) riding in a car. Items are summed to produce a total score that may range from 31 to 97, with higher scores indicating greater child sleep problems. A cut-off score of ≥ 41 has been shown to indicate a clinical sleep problem (Owens et al. [57]), and the CSHQ has previously demonstrated adequate reliability (Boergers et al. [6]). The Cronbach’s alpha for the CSHQ in the current study was 0.80.

The PROMIS-EC-SP is a 16-item parent report measure assessing child sleep problems. Parents are required to rate on a 5-point scale ranging from 1 (never) to 5 (always) the frequency with which each item applies to their child. Items are summed to produce a total score that may range from 16 to 80, with higher scores indicative of greater child sleep problems. The PROMIS-EC-SP has demonstrated excellent reliability in previous studies (α = 0.90; Lai et al. [40]). The Cronbach’s alpha of the PROMIS-EC-SP in the current study was 0.87.

#### Secondary Outcomes

*Child Anxiety*. The 28-item, parent reported Preschool Anxiety Scale (PAS; Spence et al. [71]) was used to assess child anxiety. Parents are required to rate on a 5-point scale from 0 (not true at all) to 4 (very often true), how true each item is of their child. Items are summed to produce a total score that may range from 0 to 112, with higher scores indicative of greater child anxiety. In previous studies, the PAS has demonstrated good reliability (α = 0.89; Donovan et al. [17]). In the current study, the Cronbach’s alpha was 0.89.

*Child Behaviour Problems*. The 27-item, Behaviour Scale of the Child Adjustment and Parent Efficacy Scale (CAPES; Morawska et al. [53]) was used to assess child behaviour problems. Parents are required to rate on a 4-point scale from 0 (not at all) to 3 (very much), the degree to which each item has been true of their child over the past 4 weeks. Items are summed to produce a total score that may range from 0 to 81, with higher scores indicating greater child behaviour problems. The Behaviour Scale of the CAPES has demonstrated excellent reliability in previous studies (α = 0.90; Morawska et al. [53]). In the current study the Cronbach’s alpha was 0.79.

*Parent Sleep*. The 8-item PROMIS^®^ Short Form v1.0– Sleep Disturbance 8a (PROMIS-SD; Yu et al. [87]) was used to measure parent sleep problems. Items related to the past week are presented on a 5-point Likert scale. The first item requires respondents to rate their sleep from *1 = Poor* to *5 = Very good.* The remaining 7 items require respondents to rate the degree to which each item (e.g., *My sleep was refreshing*) applies to them from 1 = *Not at all* to *5 = Very much.* Item scores are summed to produce a total score that may range from 8 to 40, with higher scores indicating greater parent sleep problems. The scale has previously demonstrated excellent internal consistency (α = 0.90; Cella et al. [11]). In the current study, the Cronbach’s alpha was 0.91.

*Parent Mental Health*. The 21-item Depression, Anxiety and Stress Scale (DASS- 21; Lovibond and Lovibond [43]) was used to assess parental mental health. The DASS- 21 consists of three, 7-item subscales assessing Depression, Anxiety and Stress. Respondents rate the degree to which each item (e.g., *I found it difficult to relax)* has applied to them over the past week on a 4-point Likert scale from *0 = Did not apply* to *me at all*,* to 3 = Applied to me to a considerable degree.* Subscale scores are derived by summing items from the subscale and multiplying by 2 (for consistency with the longer 42-item DASS). Subscale scores may therefore range from 0 to 42, with higher scores indicating greater depression, anxiety, or stress symptoms. Excellent internal consistency has previously been demonstrated for the Depression (α = 0.88) Anxiety (α = 0.82), and Stress (α = 0.90) subscales of the DASS- 21 (Henry and Crawford [34]). In the current study, the Cronbach’s alphas for the Depression, Anxiety and Stress subscales were 0.81, 0.75, and 0.83, respectively.

*Parental Self-Efficacy*. The 16-item Me as a Parent scale (MaaPs; Hamilton et al. [29]) was used to measure parental self-efficacy. Parents are required to rate their agreement with each item on a 5-point Likert scale from 1 (strongly agree) to 5 (strongly disagree). Items are summed to produce a total score that may range from 16 to 80, with higher scores reflecting greater parental self-efficacy. The MaaPs has previously demonstrated very good internal consistency (α = 0.84; Hamilton et al. [29]). In the current study, the Cronbach’s alpha was 0.91.

### Procedure

Human Ethics approval was sought and obtained as part of a larger study (Griffith University Ref No: 2023/079). Participants were recruited through social media (Facebook), kindergartens, and the Griffith University call for research, and provided informed consent to participate. Those who met eligibility criteria were provided with the online battery of T1 questionnaires through Qualtrics, after which, Session 1 of the Lights Out Online program was made available to them through a link sent via email. Participants were encouraged to complete sessions weekly and were provided with links to each subsequent session 5 days after completion of the previous session. Participants were sent a text reminder if they had not completed a session within 5 days of being sent a session link, and an email reminder after 7 days of being sent a session link. All participants were asked to complete the T2 battery of online questionnaires via Qualtrics 12 weeks post-baseline, regardless of how many sessions they had completed within that time.

### The Lights Out Online Program

Lights Out Online is a 4-session, self-directed online program for parents of children aged 3–6 years with behavioural sleep problems. It was adapted from the original group-based, 5-session, face-to-face Lights Out program, and is hosted on the authors’ (CD and SM) Momentum online platform (momentumhub.org.au), although it is not yet available to the general public. There is no therapist guidance or support provided. The content of Lights Out Online is identical to the original program in terms of the information and strategies provided to parents. The format of each session is also identical to that of the original program, with sessions mimicking face-to-face therapy. Each session begins with an agenda that is then followed by session content and the setting of homework activities. The sessions take between 30 and 60 min to complete and are designed to be engaging, with interesting graphics, interactive activities, and videos. Sessions are accessed sequentially and become available 5 days after completion of the previous session to ensure sufficient time for homework to be completed and strategies to be processed and implemented. The Lights Out Online program was compressed from 5 sessions in the original program to 4 sessions in the online format, as more time-consuming group-based activities and discussion (e.g., getting to know you activities and the sharing of homework experiences) were not included. Session 1 contains psychoeducation around sleep and sleep hygiene, while Session 2 focuses on bedtime routines and strategies to praise and reward children. Session 3 consists of strategies to manage night-time fears, while Session 4 focuses on strategies to manage misbehaviour and night-time resistance as well as parental self-care.

### Data Analysis

Summary statistics are displayed as mean (standard deviation; *SD*) for continuous data and frequency (percentage) for categorical data. Attrition analyses were conducted to compare participants who completed versus did not complete the T2 assessment, on baseline demographic and outcome variables, using independent t-tests. To investigate how the outcome variables changed over time, a series of mixed-effects linear regression models were constructed. Time was included as a 2-level (baseline and post) fixed effect. Child was modelled as a random intercept. Model assumptions, specifically approximate normality of residuals and homoscedasticity were confirmed to hold. The change from baseline to post-assessment is presented as mean difference (MD) and 95% confidence intervals (95% CI). Within-group effect sizes are summarised by Cohen’s d statistics, which were calculated as the modelled MD divided by the baseline sample SD. Analyses were conducted using Stata statistical software v14.0 (StataCorp, College Station, TX).

## Results

### Program Adherence

Participants completed an average of 3.58 out of 4 sessions. Of the 24 participants, all (100%) completed Sessions 1 and 2, 18 (75%) completed Sessions 1, 2 and 3, and 16 (67%) completed all four sessions. At T2, 18 (75%) of the total 24 participants accessed the post-intervention assessment. Of the 18 participants completing the T2 assessment, all (100%) completed Sessions 1 and 2, 14 (77.78%) completed Sessions 1, 2 and 3, and 13 (72.22%) completed all four sessions. Of the six participants who did not complete the T2 assessment, all six (100%) completed Sessions 1 and 2, four (67%) completed Sessions 1, 2 and 3, and three (50%) participants completed all four sessions.

Sessions 1 and 2 (*n* = 24) were completed in an average of 7 and 6 days respectively, while Session 3 (*n* = 18) and Session 4 (*n* = 16) took an average of 10 and 11 days respectively. Among the 16 participants who completed all four sessions, the average time to finish the program was 6.21 weeks (43.5 days), with a minimum of 21 days and a maximum of 73 days. The 18 participants who completed three sessions took an average of 4.19 weeks (29.3 days) to complete the program, with a minimum of 14 days and a maximum of 53 days. All 24 participants completed the first two sessions in an average of 2.53 weeks (17.7 days), with a minimum of 6 days and a maximum of 41 days.

### Program Acceptability

Descriptive statistics for the four discrete acceptability and satisfaction questions are provided in Table 2. Overall, of the 15 participants who completed these questions, 86.7% reported that they would tell a friend about the Lights Out Online program; 93.3% found the program at least somewhat helpful; 100% reported being at least somewhat happy with the program; and 83.3% thought the program was good or extremely good. The average acceptability and satisfaction score was 3.85 (*SD* = 0.60) out of a possible 5.

**Table 2:** Treatment Satisfaction Ratings (*n* = 15)

	*M*	*SD*	*n* (%)				
			1	2	3	4	5
			Definitely would not	Probably would not	Unsure	Probably would	Definitely would
Would you tell a friend about this program if their child had sleep problems?	4.00	0.76		1 (6.7)	1 (6.7)	10 (66.7)	3 (20)
			Extremely unhelpful	Unhelpful	Somewhat helpful	Very helpful	Extremely helpful
How helpful was this program in assisting you to manage your child’s sleep problem?	3.33	0.72		1 (6.7)	9 (60.0)	4 (26.7)	1 (6.7)
			Extremely unhappy	Unhappy	Somewhat happy	Very happy	Extremely happy
How happy were you with this program?	3.73	0.70			6 (40.0)	7 (46.7)	2 (13.3)
			Extremely bad	Bad	Okay	Good	Extremely good
Overall, I think this program was…	4.00	0.54			2 (13.3)	11 (73.3)	2 (13.3)

### Effects of the Program on Child and Parent Outcomes

*Attrition Analyses.* Given that not all participants completed the T2 assessment, differences in T1 demographic and outcome variables between those who were retained at T2 versus those who were not retained were assessed. As detailed in Table 3, there were no significant differences in demographic or outcomes variables between T2 completers and non-completers, with the exception of parent anxiety. Parents who did not complete the T2 assessment (*n* = 6, *M* = 10.33, *SD* = 8.14) scored significantly higher on the DASS-Anxiety subscale than those who completed the T2 assessment battery (*n* = 18, *M* = 4.00, *SD* = 3.88), *t*(22) = 2.60, *p* = 0.016.

**Fig. 2:**
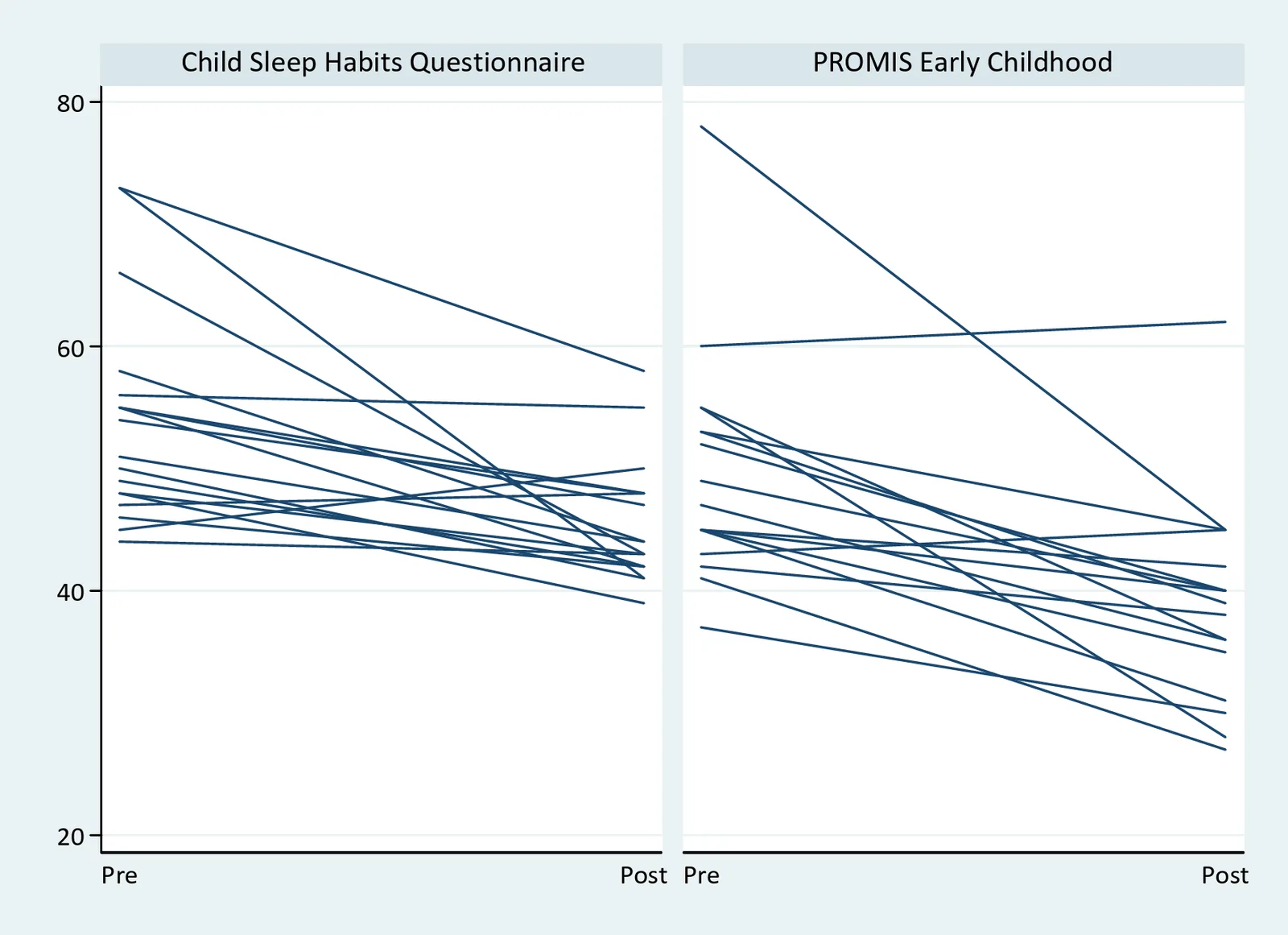
Individual changes from T1 to T2 on child sleep measures (CSHQ and PROMIS-EC-SP)

*Primary Outcomes: Child Sleep.* Table 4 displays the means and standard deviations of all variables at both time points, as well as within-group change over time. Figure 2 plots the data for the primary outcomes graphically. In support of the hypotheses, and as can be seen in Table 4, significant improvements in child sleep were evident on the CSHQ (MD = − 9.0; 95% CI: − 12.8, − 5.3) and PROMIS-EC-SP (MD = − 10.8; 95% CI: − 14.7, − 6.9).

**Table 3:** Differences in demographic and outcome variables between those who completed the T2 assessment and those who did not

Variable	Did not complete T2 (*n* = 6) *M (SD)*	Completed T2 (*n* = 18) *M (SD)*	*P*-value
Parent age	35.00 (3.0)	36.56 (4.4)	0.43
Child age	4.00 (1.3)	4.17 (1.0)	0.75
Number of children in household	2.33 (1.0)	1.94 (0.5)	0.24
CSHQ	56.3 (7.5)	54.1 (9.8)	0.58
PROMIS-EC	49.0 (6.5)	49.6 (9.2)	0.88
PAS	29.2 (17.6)	35.0 (17.2)	0.48
CAPES behavioural difficulties	32.2 (10.2)	33.2 (19.9)	0.87
DASS- 21 Depression	9.3 (9.9)	5.0 (4.4)	0.15
DASS- 21 Anxiety	10.3 (8.1)	4.0 (3.9)	0.02
DASS- 21 Stress	20.3 (10.4)	15.6 (7.4)	0.23
MaaPs Total	60.0 (4.4)	59.7 (10.2)	0.95
PROMIS-SD	25.3 (8.0)	22.8 (7.3)	0.47

Note. CSHQ = Child Sleep Habits Questionnaire. PROMIS-EC = PROMIS Early Childhood Parent Report Sleep Problems measure. CAPES = Child Adjustment and Parent Efficacy Scale. DASS = Depression Anxiety Stress Scales. MaaPs = Me as a Parent Scale. PROMIS-SD = PROMIS Adult Sleep Disturbance measure

*Secondary Child Outcomes*. As Table 4 suggests, and in support of the hypotheses, significant improvements in child anxiety and behaviour from T1 to T2 were evident on the PAS (MD = − 10.4; 95% CI: − 15.1, − 5.7) and CAPES (MD = − 6.1; 95% CI: − 10.0, − 2.2), respectively.

*Secondary Parent Outcomes.* In support of the hypotheses, and as can be seen in Table 4, significant improvements in parent sleep from T1 to T2 were observed on the PROMIS-SD (MD = − 3.4; 95% CI: − 6.4, − 0.4), with similar significant improvements evident in parental self-efficacy on the MaaPs (MD = 5.5; 95% CI: 1.9, 9.1) and parent anxiety on the DASS-Anxiety subscale (MD = − 1.6; 95%CI: − 3.1, − 0.1). Non-significant decreases were observed for parent depression and stress on the DASS-Depression (MD = − 1.0; 95%CI: − 3.4, 1.5) and DASS-Stress (MD = − 3.5; 95%CI: − 7.3, 0.3) subscales.

**Table 4:** Change in key outcome variables from pre- to post-intervention

Variable	Baseline		12-week post-baseline		Estimated change from pre- to post-intervention		
	*n*	*M (SD)*	*n*	*M (SD)*	*MD (95% CI)*	*p*	*Cohen's d*
Child Sleep Problems							
CSHQ	24	54.6 (8.4)	18	45.4 (5.0)	–9.0 (–12.8,–5.3)	< 0.001	–1.08
PROMIS-EC	24	49.5 (8.5)	17	38.8 (8.3)	–10.8 (–14.7,–6.9)	< 0.001	–1.28
Child Anxiety Problems							
PAS	24	33.5 (17.1)	16	25.6 (14.8)	–10.4 (–15.1,–5.7)	< 0.001	–0.61
Child Behavioural Difficulties							
CAPES behavioural difficulties	24	30.0 (12.0)	16	24.8 (8.3)	–6.1 (–10.0,–2.2)	0.002	–0.51
Parent Psychological Distress							
DASS- 21 Depression	24	6.1 (6.3)	16	4.5 (4.8)	–1.0 (–3.4, 1.5)	0.42	–0.16
DASS- 21 Anxiety	24	5.6 (5.8)	16	3.00(3.3)	–1.6 (–3.1,–0.1)	0.04	–0.27
DASS- 21 Stress	24	16.8 (8.3)	16	13.3 (9.1)	–3.5 (–7.3, 0.3)	0.07	–0.42
Parental Self-Efficacy							
MaaPs Total	24	59.9 (9.0)	16	65.1 (9.3)	5.5 (1.9, 9.1)	0.003	0.61
Parent Sleep Problems							
PROMIS-SD	24	23.4 (7.4)	16	20.0 (6.8)	–3.4 (–6.4,–0.4)	0.03	–0.46

*Note. *CSHQ = Child Sleep Habits Questionnaire. PROMIS-EC= PROMIS Early Childhood Parent Report Sleep Problems measure. CAPES= Child Adjustment and Parent Efficacy Scale. DASS= Depression Anxiety Stress Scales. MaaPs= Me as a Parent Scale. PROMIS-SD= PROMIS Adult Sleep Disturbance measure. MD = Mean Difference. CI = Confidence Interval

## Discussion

This study sought to determine the feasibility of the Lights Out Online program, a parent-focused, self-directed online program targeting behavioural sleep problems in children aged 3–6 years. Adherence to, and acceptability of, the program were assessed, together with the program’s potential to positively impact child (sleep, anxiety and behaviour problems) and parent (sleep, parental self-efficacy and mental health) outcomes. Parental adherence to, and satisfaction with, the program was found to be high. Furthermore, significant improvements from baseline (T1) to 12-weeks post-baseline (T2) were evident for child sleep, anxiety and behaviour problems as well as for parental self-efficacy, sleep and anxiety. Significant improvements were not evident for either parent depression or parent stress from T1 to T2.

With respect to adherence, parents completed an average of 3.58 out of 4 sessions. All parents completed Sessions 1 and 2, with 75% completing Session 3 and 67% completing Session 4. In addition, parents completed sessions in a relatively timely fashion, completing Sessions 1 and 2 in an average of 6–7 days after they became available, Session 3 within 10 days of it becoming available, and Session 4 within 11 days once it was available. Furthermore, of the 16 participants who completed all 4 sessions, the average time to complete the program was 6.21 weeks (43.5 days). Although session completion rates were poorer for Lights Out Online compared to the original program where all parents completed all 5 sessions, they are superior to other entirely self-directed parent programs targeting child difficulties (e.g., Morgan et al. [54]). Potential explanations for why 33% of participants in this study failed to complete all online sessions include program dissatisfaction, receiving sufficient dose and/or ‘getting enough of what they need’, early improvement, and less commitment due to lack of a therapist and therefore therapeutic alliance. Although this trial was not large enough to examine predictors of, and reasons for, non-adherence, future larger trials should prioritise these objectives to determine who might best benefit from unguided online programs, and to develop strategies that may be put in place to encourage parents to complete a greater number of sessions.

Relatedly, 25% of participants did not complete the T2 assessment battery, demonstrating significant attrition in this trial. Again, trials of online programs are plagued by high levels of attrition (Hall and Bierman [28]), the reasons for which remain unclear, but may be similar to the reasons for non-adherence. Given the importance of retention to the scientific integrity of a trial, future research should attempt to determine the reasons underpinning high attrition in studies examining online programs, so that strategies to enhance retention can be incorporated into trial designs.

With respect to treatment satisfaction, although parents were generally satisfied with the program, there was clearly room for improvement. We have recently conducted interviews with parents, with the aim of gaining insight into the user experience, and to seek feedback and recommendations on potential modifications to the program (Etel et al., 2025). The consumer-informed program refinements have been made to Lights Out Online, with the resulting ‘Version 2’ being trialled in a RCT that is currently being conducted. It is hoped that by responding to the parent voice, parent satisfaction (and perhaps adherence and retention) with the program will be higher for Version 2.

Results pertaining to the demonstrated effects of *Lights Out Online* on child and parent outcomes should be taken with caution given the very small sample size and absence of a control group. However, this study provides preliminary evidence in support of the program’s potential to improve child sleep, anxiety and behaviour problems, as well as parental self-efficacy, sleep and anxiety. That a significant improvement in child sleep problems was evident is consistent with the positive child sleep outcomes of the few studies on fully self-guided online parenting behavioural interventions that have included (but were not limited to) young children (Brandhorst et al. [8]); Hiscock et al. [36]); Schlarb and Brandhorst [67]). The Lights Out Online program was developed from a face-to-face, small group program with demonstrated efficacy in reducing child sleep problems (Donovan et al. [17]). It has been proposed that there are many advantages of developing new online interventions from pre-existing psychological treatments with proven efficacy in other delivery modalities, particularly when the original content was derived from a well-researched theoretical basis (Bornstein et al., 2022). The current findings are in line with this assertion and provide proof of concept that the Lights Out Online program may be effective in treating sleep problems in young children.

Consistent with the findings of Donovan et al. [17]), significant reductions in both child anxiety and child behaviour problems were evident following parental participation in the Lights Out Online program, suggesting the potentially broader impact of *Lights Out Online* on other child outcomes. The current study is one of the few, other than the one conducted by Donovan et al. [17]) testing the face-to-face version of *Lights Out*, to demonstrate significant effects of a child sleep program on outcomes other than sleep, and the only one other than Donovan et al. [17]) to show effects on child anxiety in particular. Given the very high prevalence rates of both anxiety and behavioural problems among preschool age children, and the myriad problematic consequences associated with them, demonstration that a 4-session sleep program may potentially produce significant reductions in sleep, anxiety and behavioural problems is exciting.

Online programs for both preschool anxiety (e.g., Donovan and March [16]) and behavioural problems (e.g., Sanders et al. [66]) have been developed and shown to be efficacious. However, they are generally several sessions in length and focus exclusively on the target problem. That the Lights Out Online program was able to demonstrate reductions in anxiety and behaviour problems by including only one session targeting each construct in the context of bedtime, demonstrates the potential efficiency of the program, and its ability to empower parents by successfully teaching them important skills that they can generalise to other child problem areas. Furthermore, that broader child impact can be evidenced with an online program that requires no therapist involvement, has significant public health implications if delivered at scale, by allowing free, convenient and flexible access to all families with an internet connection.

The finding that parental self-efficacy was significantly improved following the program is consistent with previous research by Brandhorst et al. [8]), who found that a parent-led online behavioural sleep intervention enhanced the parental self-efficacy of parents of children aged 6 months to 4 years of age. Parental self-efficacy captures the domains of parental confidence and subjective competence, and the Lights Out Online program is designed to provide parents with new parenting skills to help facilitate better child sleep. Thus, when parents observe a reduction in their child’s sleep problems via changes in their parenting, their sense of confidence and competence as a parent may increase. Given that research has demonstrated the important role that parental self-efficacy plays in child sleep, parenting behaviours, family functioning and parent wellbeing (Fang et al. [21]); Lesniowska et al. [42]); Werner et al. [82], the results of the current study are particularly encouraging, and demonstrate that the Lights Out Online program may provide benefits to the family that are above and beyond improving child outcomes.

In terms of parent sleep, the current study observed a significant improvement from T1 to T2. This finding is consistent with extant previous correlational research suggesting a relationship between sleep problems in young children and parent sleep quality and quantity (Boergers et al. [6]; Smedje et al. [70]). Indeed, it makes intuitive sense that when children do not sleep, parents do not sleep, and that when children do sleep, parents are also provided with better opportunity to do so.

With respect to parental psychological distress, the results suggested significant reductions in parent anxiety symptoms, but not depression or stress symptoms, from T1 to T2. To our knowledge, this is the first study to demonstrate a significant reduction in parent anxiety symptoms following an online program for preschool sleep problems, and is consistent with literature connecting child sleep problems with higher parent anxiety (Schultz et al. [68]). However, it is important to note that average baseline levels of anxiety and depression symptoms in this sample were within the normal range (see Lovibond and Lovibond [43])). On the other hand, the lack of findings for parental stress are somewhat surprising given that mean baseline levels of stress in this sample were in the mild range while mean T2 levels of stress were within normal range (see Lovibond and Lovibond [43]), and interactions between parental self-efficacy and parent stress are well documented in the literature (Albanese et al. [1]). It may be that factors other than child sleep problems were contributing to parental stress in the current sample, or that improvements in stress will manifest when parents have had more time to implement the strategies they have learned. Future research with a larger sample should further investigate factors contributing to parental stress, and provide longer term follow-ups to determine whether parental stress and depression are improved following treatment for child sleep problems.

### Limitations and Future Directions

The current study provides a novel contribution to the literature through assessing the feasibility of a fully online, self-directed, parenting intervention designed *specifically* to address the types of behavioural sleep problems experienced by 3–6 year-old children. The study examined treatment adherence, acceptability, and a number of outcomes other than child sleep, using psychometrically sound measures. However, the study was not without limitations. Most serious among the limitations of this study was that the sample size was very small and there was no control group, thus making it impossible to conclude with any certainty that the observed improvements in child and parent outcomes were due to the Lights Out Online program rather than natural developmental changes, regression to the mean, or external factors. Furthermore, the small sample size made it impossible to determine predictors of outcome, including whether timely completion of sessions was associated with enhanced treatment outcome. Clearly, an RCT with a larger sample size and a control group is required to adequately assess the efficacy of *Lights Out Online* with respect to sleep and broader child and parent outcomes. Indeed, this small, uncontrolled study was conducted ahead of a larger RCT (currently in progress), to assess feasibility and potential efficacy, iron out any program bugs, and make modifications to the program based on parent feedback. The RCT in progress is therefore assessing *Lights Out Online V2* as a result of this important preliminary feasibility trial.

Another limitation of the study was the reliance on parent-report of child outcomes at the expense of objective, observational, and/or diagnostic interviews with a clinician. It cannot be ruled out for example, that improvements in parent sleep may have led to more positive parent reporting of child anxiety and behaviour problems. Future research should consider more objective and/or observational measures to ensure accurate assessment of child outcomes, and should include diagnostic interviews so that the program’s ability to reduce clinical levels of these disorders can be determined.

Not including additional, longer-term follow-up assessments to determine maintenance of treatment effects was another methodological flaw of this study, making it difficult to ascertain whether the results achieved were maintained over a longer time period. Although the present study was an open pilot trial, an additional assessment point would have allowed us to determine the temporal stability of treatment effects. Finally, the sample was relatively homogenous, with the majority of participants being female, highly educated and of relatively high socioeconomic status. Future research should strive for a more heterogenous sample of parents to ensure generalisability of the results.

### Implications and Conclusion

This preliminary study provides a novel and constructive contribution to the research, providing preliminary support for the feasibility of an online, parent-led behavioural intervention for child sleep problems. That a four-session, online program can potentially produce broader behavioural, health and mental health outcomes amongst these youngsters and their families, has enormous public health implications if the program is delivered at scale. Given the potential for online programs to reach all families with a computer, the task going forward will be to replicate and extend these findings with stronger methodological designs, and then to ensure that programs such as this are disseminated as widely as possible, so that as many families as possible may benefit.

## Data Availability

Data is provided within the manuscript or supplementary information files.
